# Leading through the pandemic: Experiences of diagnostic radiography middle managers during the COVID-19 pandemic

**DOI:** 10.4102/hsag.v31i0.3343

**Published:** 2026-07-31

**Authors:** Yolanda Le Roux-Arries, Valdiela Daries, Heidi Thomas

**Affiliations:** 1 Department of Medical Imaging and Therapeutic Sciences, Faculty of Health and Wellness Sciences, Cape Peninsula University of Technology, Cape Town, South Africa

**Keywords:** diagnostic radiography manager, radiography managers, experiences of managers, COVID-19 pandemic, private radiology practice

## Abstract

**Background:**

Seven years since the onset of the global coronavirus disease 2019 (COVID-19) pandemic, healthcare delivery, particularly within radiology and imaging services, has been reshaped. Amid the surge of COVID-19 infection rates, diagnostic radiography middle managers (DRMMs) were tasked with implementing new protocols, managing increased imaging demands and leading teams through uncharted territory. The exposed gaps in middle management crisis preparedness during the global health emergency highlighted the need for support structures within the imaging sector.

**Aim:**

This article focuses on the experiences of DRMMs during the COVID-19 pandemic and the management strategies they applied in response to the global health emergency.

**Setting:**

The study was conducted at a private radiology practice operating at various imaging branches in the Western Cape and KwaZulu-Natal in South Africa.

**Methods:**

A qualitative study was conducted using a case study design. Eleven DRMMs were recruited through purposive sampling. Semi-structured interviews were conducted and recorded via the Zoom online platform. Manual coding was performed, and the data were analysed using thematic analysis.

**Results:**

Three main themes were identified: adapting to change, challenging environment and managerial growth amid the pandemic. The DRMMs expressed the various operational management challenges they experienced, the uncertainties and doubts they had to navigate and the new skills and abilities they developed.

**Conclusion:**

The study participants experienced feeling out of control and unprepared at the start of the pandemic, but they surmounted the difficulties and grew from the experience. The recommended frameworks are designed to provide support to DRMMs during future global health emergencies.

**Contribution:**

The study demonstrated that better-structured support mechanisms for DRMMs should be in place to safeguard radiology staff members from potential adverse effects.

## Introduction

A manager is responsible for planning, organising, leading and controlling resources to achieve organisational goals effectively (Lloyd & Aho 2020:7). Within the healthcare setting, managers ensure that operations run smoothly while maintaining quality, efficiency and staff well-being. Radiology departments offer diagnostic and therapeutic services that involve complex technologies and multidisciplinary teamwork and are integral to patient care (Chau 2024:S10). In diagnostic radiography, the manager oversees imaging services, staff coordination, workflow management and adherence to safety and ethical standards (McFadden et al. 2022:S73). In the study context, diagnostic radiography middle managers (DRMMs) are employees of a radiology practice who supervise frontline radiography staff members and are responsible for the general management of the imaging branch. This position is also referred to or known as a Head of Department (HOD). At the start of the coronavirus disease 2019 (COVID-19) pandemic in March 2020, DRMMs had to ensure the continuous delivery of essential radiological examinations and procedures. As the coronavirus affected the lungs of infected individuals, radiographic chest X-rays (CXR) and computed tomography (CT) examinations played a crucial role in the diagnosis of severe acute respiratory syndrome coronavirus 2 (SARS-CoV-2) (Akudjedu et al. 2020:8; Aljondi et al. 2020:52; Hundah, Sibiya & Khoza 2024:5; Jorge & Fridell 2021:378). As described by Wu et al. (2020:219), CXR and CT examinations usually revealed bilateral pneumonia (in 75% – 98% of cases) with multiple mottling and ground-glass opacity. Akyurt (2021:1) clarified that the requests for CXR and CT examinations as diagnostic imaging tools increased because these imaging examinations became the preferred imaging in diagnosing COVID-19 pneumonia. As the pandemic progressed, the various branches of the research site experienced an increase in requests for CXR and CT imaging of the chest owing to their demonstrated value in assessing lung patterns in patients infected with the coronavirus. As described by Akudjedu et al. (2020:8), to facilitate changes in work patterns during the coronavirus pandemic, managers in hospitals and radiography departments played an integral role in ensuring all aspects of operations management were adhered to. Tay et al. (2020:469) expressed that the need for leadership with the foresight to anticipate potential operational issues became crucial to managing operations in the face of the pandemic. New operations management adjustments at the study site that had to be implemented included dividing radiographers into two groups: one group on duty and the other off duty. This was to restrict staffing levels across different modality units. Services such as mammography, bone mineral density scanning, fluoroscopy and magnetic resonance imaging (MRI) examinations that were deemed non-urgent were suspended. Diagnostic radiography middle managers had to adapt to the sudden operational adjustments that constantly changed as the pandemic progressed. Since the onset of the pandemic, a substantial body of published research has reported on the effects of the pandemic on nurse practitioners and radiographers, with limited available literature on the management strategies employed by DRMMs during the pandemic (Aljondi et al. 2021; Blake et al. 2020; Chinene 2023; Hundah et al. 2024; Jorge & Fridell 2021; Lewis & Mulla 2021; Naylor et al. 2022; Ogolodom et al. 2020; Ruiz et al. 2021; Sehularo et al. 2021; Tariq et al. 2021; Williams et al. 2021; Zervides et al. 2020). From the researcher’s experience, various new working requirements were implemented by DRMMs, and radiographers had to learn how to adapt to the latest health and safety regulations to safely work with highly infectious patients. The need for a change in workflow, facilitated by DRMMs, was necessary to minimise the risk of cross-infection within the radiology department and the hospital. In addition to their daily duties, DRMMs had to put policies, plans and protocols in place while at the same time experiencing tremendous uncertainty. Therefore, this study aimed to explore the experiences of DRMMs during the COVID-19 pandemic and the management strategies they applied.

## Research design and methods

### Research setting

The study was conducted among 11 DRMMs working across various branches of a private radiology practice that operates in the Western Cape and KwaZulu-Natal. The private radiology practice is a leading diagnostic imaging group offering a wide range of imaging modalities, including X-rays, CT, MRI and ultrasound. The radiology practice provides patient-centred services across hospital-based and outpatient settings, with emergency radiology support and 24-h operations at key hospital sites.

### Research design

This study employed an exploratory and descriptive qualitative approach using a case study design, underpinned by an interpretivist perspective. Lester, Cho and Lochmiller (2020:96) regard qualitative data analysis as a means of bringing meaning to a dataset through conversational data, observations and interviews, whereas Alharahsheh and Pius (2020:41) argue that interpretivism enables researchers to explore further depth and insights into individual experiences through formal discussion and interviews. One-on-one semi-structured online interviews were conducted to gain an in-depth understanding of the experiences of DRMMs. Furthermore, the video recordings allowed the researcher to observe the non-verbal cues such as body language, facial expressions and gestures exhibited by the participants. By doing so, the researcher could delve deeper into the meanings and underlying understanding of the answers provided, thus gaining a more comprehensive perspective of the participants. Data analysis was conducted using thematic analysis to identify themes or patterns in the data that were important or of interest in addressing the research question, which was:

*‘What were the experiences of DRMMs working at a private radiology practice within South Africa during the COVID-19 pandemic?’*

### Study population and sampling strategy

The recruitment strategy involved inviting all DRMMs to participate in the study. The study population consisted of 11 DRMMs who worked at the private radiology practice between March 2020 and April 2022, selected through purposive sampling. As described by Ahmad and Wilkins (2024:3), purposive sampling involves deliberately selecting participants who share specific qualities to answer a research inquiry about a phenomenon. Consequently, the participants of this study were not randomly selected, but this enabled the inclusion of DRMMs who could provide rich and detailed explanations of their experiences during the COVID-19 pandemic.

### Inclusion criteria

- Diagnostic radiography middle managers who were employed at the various imaging branches of the radiology practice during the South African national state of disaster period, March 2020 to April 2022.
- This timeframe indicates the start-to-end dates of the South African national state of disaster (Department of Co-Operative Governance and Traditional Affairs, South Africa, 2022). This specific timeframe and inclusion criteria were selected, as the researcher wanted to include the experiences of as many DRMMs as possible who were employed as radiography middle managers at the research site during the COVID-19 pandemic.

### Exclusion criteria

Diagnostic radiography middle managers who were not employed at the specific private radiology practice during the period of the South African national state of disaster.

### Data collection

Permission was obtained by sending an email to the managing committee of the radiology practice, requesting authorisation to communicate with participants via their official work email addresses. After approval was granted, the researcher began recruiting the prospective research participants. The researcher compiled an email which included the permission letter from the practice, the study information letter, and the participant consent form, inviting DRMMs to participate in the study. The email was distributed to the work email addresses of all the DRMMs employed at the radiology practice and who were employed during the inclusion criteria period. The researcher reserved 1 month for DRMMs to respond to the study invitation. After the initial 3 weeks had passed, a reminder email was sent to potential participants. The researcher remained mindful of the participants’ right to decline to participate in the study; therefore, no further contact was made with the DRMMs after the initial reminder was sent. Eleven DRMMs voluntarily consented to participate in the study. Informed consent was revisited before the commencement of the data collection process. Data collection was in the form of one-on-one online video-recorded semi-structured interviews. The semi-structured interviews provided the researcher with the opportunity to have one-on-one conversations with each participant, with the added advantage of using a blend of closed- and open-ended questions. Before the commencement of the interview, a secure link which allowed access to the interview was sent to participants’ work e-mail addresses. These accounts were protected by phishing software to avoid unauthorised access to work e-mails. Participants were informed that they could withdraw their consent at any time if they felt uncomfortable and no longer wished to participate. Because of familiarity with the Zoom Communication application, the researcher chose to use this platform to conduct the semi-structured interviews. The Zoom platform also provides password protection for confidentiality. Additionally, the recording automatically saved the interview into two files: an audio-only file and a combined audio-video file. The interviews were conducted at a time that was convenient for both the participant and the researcher. Because of work schedules and participant availability, data collection for this study was conducted over two non-consecutive months, December 2022 and March 2023. This timeframe was influenced by work schedules and participant availability. Interviews lasted between 22 min and 50 min per interview. During data collection, the researcher was cognisant of data saturation applicable to qualitative research. Guest, Namey and Chen (2020:5) explain saturation as the point reached during data analysis when incoming data produces little or no new useful information relative to the study objectives. Similarly, Weller et al. (2018:1) assert that sample size determination for open-ended questions relies primarily on finding the point where little new information is obtained. In this study, the researcher observed that participants began recalling information at different stages of the interview. This valuable data, which the researcher believed would be useful as part of the study findings, led the researcher to continue conducting interviews to gather rich, unique insights. Consequently, all 11 participants who volunteered for the study were interviewed.

### Data analysis

Data analysis was conducted using thematic analysis. The researcher followed the six steps of the thematic analysis framework as described by Clarke and Braun (2013:3). These steps were:

- *Familiarisation with the data*: The repetitive reading of the data allowed the researcher to become familiar with and gain a deeper understanding of the data.
- *Generated initial codes*: By using the Excel application, the researcher worked through each segment of the text that seemed relevant by writing the phrases in a column next to the extract.
- *Search for themes*: Similar concepts were identified and grouped into initial categories, which were combined to form themes.
- *Review themes*: After reviewing the initial themes, the researcher refined them into more meaningful and clearer themes.
- *Define and name themes*: Themes were named by creating definitions that embodied a meaningful and comprehensive description of the subject matter.
- *Writing up*: Lastly, the report was produced, which incorporated verbatim participant quotations to enhance the authenticity of the data and validate the themes that emerged.

### Measures of trustworthiness

To ensure rigour in the research process, the principles of trustworthiness, namely credibility, dependability, transferability and confirmability, were adhered to. Extensive direct quotations from the written responses of the participants were used to give the reader a sense of the authenticity of the responses and the generation of the themes. The researcher was cognisant of bracketing her views and opinions. Careful attention was given to describing the experiences of the participants by using direct quotations. As explained by Ayton (2023:233), research is considered trustworthy when members of the research community are confident in the study’s methods, the data and their interpretation. Ayton (2023:236) further emphasised that quotes from participants are used to demonstrate that the themes are generated from the data. Reflexivity was also acknowledged, and as described by Berkovic (2023:242), it involves researchers acknowledging and disclosing their involvement in research to understand their impact on it. Therefore, to outline the research process that was followed, a diary was kept, and reflective notes were made during interviews. In this qualitative study, details were provided of the context to make transferability possible by providing a dense description of the study participants, methodology and findings of the study, should researchers wish to do so. The researcher kept a record of all the research steps, the audio-video recordings, personal reflections and participant transcriptions. Additionally, an audit trail is available whereby the researcher documented all the steps taken and the decisions made regarding the analysis and coding of the data.

### Ethical considerations

Ethical permission to conduct the study was obtained from the relevant Research Ethics Committee of the Faculty of Health and Wellness Sciences at the university where the researcher was enrolled (REC Approval Reference no: CPUT/HWS-REC 2022/H13). Permission to invite the middle managers from the selected private practice branches and to contact them via their work email addresses was obtained from the managing committee of the relevant private radiology practice. Participants received a participant information sheet explaining all details related to the study before partaking in the study and were assured that participation was completely voluntary and that all information gathered was regarded as private and confidential. All participants were informed that they could withdraw their consent at any time during the study. The researcher had a confidentiality agreement with the transcriber, and only the audio recordings were sent via a secure email link to the personal email account of the transcriber. Participants were not coerced, nor were they compensated to participate in the study.

## Findings

The work experience of participants varied, with some having less than 5 years of experience in the role as DRMM, while others had more than 10 years. Table 1 provides a summary of the demographic details of the study participants, with MMP1, MMP2 and so forth referring to middle manager and participant number.

**TABLE 1:** Demographics of participants.

Middle Manager and Participant Number	Years of experience as a diagnostic radiographer	Years of experience as a DRMM	Number of staff members being managed
MMP1	6–10	Less than 5 years	Between 21 and 30
MMP2	11–15	Less than 5 years	Between 21 and 30
MMP3	20+	More than 10 years	More than 30
MMP4	20+	More than 10 years	More than 30
MMP5	16–20	Less than 5 years	Between 21 and 30
MMP6	20+	More than 5 years, but less than 10 years	Between 21 and 30
MMP7	20+	Less than 5 years	More than 30
MMP8	20+	Less than 5 years	More than 30
MMP9	20+	Less than 5 years	Between 21 and 30
MMP10	11–15	Less than 5 years	Between 21 and 30
MMP11	20+	More than 5 years, but less than 10 years	Between 21 and 30

DRMM, diagnostic radiography middle managers.

The interview guide facilitated the semi-structured interviewing process by ensuring consistency of questions across participants, while allowing flexibility and in-depth exploration of participant responses. Table 2 provides an overview of the interview guide questions.

**TABLE 2:** Semi-structured interview guide.

No.	Interview questions
1	Please describe how you managed or coped with your responsibilities at the start of the pandemic.
2	Reflecting on your role as a middle manager, please describe the experiences you may have had.
3	Please describe some of the measures you had to put in place in the radiology department in response to the pandemic. Probe: Consider staffing and operations management factors.
4	What type of challenges (staff-related and/or operations-related) did you experience as a middle manager during the COVID-19 pandemic?
5	What new work practices or policies were implemented during the pandemic that you had to manage?
6	Please elaborate on your experiences in overcoming any challenges that you may have experienced as a middle manager.
7	Please describe any new practices, such as protocols or policies, that you had to implement during the pandemic. Probe: Elaborate on your role and experiences in implementing these measures.
8	Please describe some of the added responsibilities assigned to you during the COVID-19 pandemic.
9	Please explain how you managed the added responsibilities brought about by the pandemic. Probe: elaborate on the coping strategies you used.
10	Please describe the type of support you received from top management that assisted you in fulfilling your role as a middle manager.
11	Please describe the type of support you received from radiographers that assisted you in fulfilling your role as a middle manager.
12	Please describe any new skills you acquired during the pandemic that you will continue to use as a middle manager going forward.
13	Please describe any personal and professional lessons you have learned during the pandemic that could be of value to other middle managers in future.

COVID-19, coronavirus disease 2019; No., number.

**FIGURE 1:**
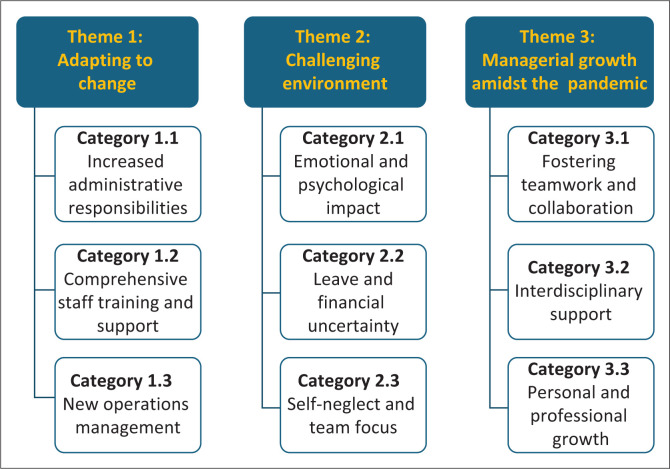
Themes and categories.

Following the online semi-structured interviews, three main themes and various categories were identified. Figure 1 presents a comprehensive summary of the themes and categories that emerged from the data analysis process.

### Theme 1: Adapting to change

This theme describes the sudden operational adjustments, development of new protocols and necessary changes that had to be implemented to maintain and prioritise imaging services during this crisis period.

#### Category 1.1: Increased administrative responsibility

Participants expressed their disbelief at the amount of administrative work they had to document and keep records of. The workload was further complicated by the need to conduct meetings, maintain accurate charts, documentation and the billing of new medical aid codes, all while adapting to the ever-changing protocols and procedures. Verbatim quotations demonstrate the increased administrative responsibilities:

‘Added duties, was tracking, so if someone came into contact, the endless paperwork of who was your first possible contact and how long you were in contact with that person, to then classify if you’re going to go into isolation or not.’ (MMP1)

‘From my point of view, the admin was endless, and the temperature checks, and that annoying thing when somebody was sick, you had to do that contact tracing form. That was my absolute worst.’ (MMP3)

Responses from participants in the current study reported increased administrative responsibilities, including recordkeeping, contact tracing and enforcing new protocols.

#### Category 1.2: Comprehensive staff training and support

The statements made by DRMMs suggest that they were ill-prepared to manage their personnel and prepare their departments to adhere to all the newly implemented guidelines. They shared their efforts to ensure that the radiology staff had a comprehensive understanding of the virus’s spread and effective measures to prevent its further dissemination. They had to educate their staff members on how to apply safe infection control measures. Diagnostic radiography middle managers found it challenging to educate radiographers on all aspects; a few mentioned:

‘I had to arrange handwashing workshops with the infection control sister from the hospital with the different teams on duty.’ (MMP9)

‘I felt their fears with them. Every time there was a change to the policy, I was here with them, discussing it, talking through what the challenges are, talking through how we are going to implement what we need to implement, taking the ideas when they come to me with an idea and seeing how we can incorporate what they suggest into what we have to do as part of our protocol or policy.’ (MMP5)

Diagnostic radiography middle managers had to conduct training sessions on how to safely clean the machines to prevent equipment damage, facilitate the safe disposal of used personal protective equipment (PPE) to ensure staff members did not get infected with the coronavirus. They also had to conduct demonstration sessions for staff members to guide them on the correct procedure to safely discard contaminated COVID-19 waste.

#### Category 1.3: New operations management

Diagnostic radiography middle managers mentioned that they had to adapt the staffing rosters more frequently and assign different personnel to specific tasks to manage the workflow. Strict entry protocols and designated spaces for infected patients were implemented to safeguard uninfected individuals and streamline infection control within the department. They also expressed that they had to adapt to using different platforms for daily meetings, which facilitated communication and helped them navigate and brainstorm the new workflow with peers and top management. Participants mentioned the following:

‘We were heavily short-staffed compared to what our normal complement of staff is. So from an operations perspective, we had to limit bookings to what we could cope with.’ (MMP5)

‘We had questionnaires at the door when people entered the department, sanitising their hands, ensuring that anybody who entered the department had a mask on, so basically a screening procedure. We also had separate chairs, so people didn’t sit next to each other. To limit seating, we stopped allowing relatives into the departments, ensuring that only those who needed to undergo examinations were present.’ (MMP4)

‘Our meetings were on a different platform, so we communicated via virtual meetings.’ (MMP8)

To ensure their departments adhered to national requirements of limiting the spread of the virus while maintaining adequate staffing for necessary imaging services, DRMMs had to alter staff duty arrangements daily. This was especially important if staff members became infected with the virus.

### Theme 2: Challenging environment

Given the uncertain work environment caused by the pandemic, the DRMMs in this study prioritised organisation and strategy development to effectively manage new challenges. They acknowledged that radiographers were at the forefront of the pandemic and were, therefore, directly exposed to the virus. It was paramount for DRMMs to comprehend the anxiety and apprehension radiographers experienced while rendering imaging services. A few DRMMs mentioned that they felt responsible for their staff members’ well-being.

#### Category 2.1: Emotional and psychological impact

Increased stress, exhaustion and being overwhelmed with all their responsibilities were emotions expressed by study participants. Despite feeling emotionally drained themselves, one DRMM compared herself to a lighthouse that had to be there for her team during this difficult time. While some DRMMs reported feeling mentally fatigued, others felt guilty for not being directly exposed to the infected patients. Many struggled to handle the stress and found it difficult to express their feelings:

‘So the pressure was quite high on the middle managers at that time. And to understand the virus and the effect it had on the staff, mentally and obviously, their health. So if I look back, because of that unknown and fear we had obviously, we as middle managers had to stay calm and be more focussed to be there for the staff 24/7.’ (MMP8)

‘I must admit I did feel overwhelmed with the responsibility; it was tough. I found that there were times when our own work was expected to carry on, but we had all this additional stress. It would’ve been nice if we could’ve had a debriefing at the end of all of this. Just a space where people can feel safe and talk about what they went through.’ (MMP11)

The DRMMs observed fear in their staff members and adopted protective behaviours and felt compelled to maintain an appearance of confidence and optimism to reassure and support their teams amidst uncertainty.

#### Category 2.2: Leave and financial uncertainty

Diagnostic radiography middle managers discussed alternative financial measures implemented by the radiology practice management in response to the crisis period’s impact on imaging operations. Diagnostic radiography middle managers also underscored the fact that salaries were being reduced because of reduced patient volumes, unpaid leave periods, and the absence of overtime compensation:

‘The staff battled with their financial stress and the salary cuts and the constraints that they have at home – where partners have lost their jobs.’ (MMP9)

‘So a month, we had ten, each employee had ten unpaid leave days.’ (MMP2)

‘We were rostered one week on, one week off. So that made it quite a bit difficult financially and physically.’ (MMP10)

Diagnostic radiography middle managers in the current study perceived uncertainty and a disconnect between financial cutbacks and the continued workload in radiology departments.

#### Category 2.3: Self-neglect and team focus

Study participants observed the exhaustion of radiographers and experienced private emotional breakdowns themselves, as they did not want to appear vulnerable. Diagnostic radiography middle managers prioritised their team’s emotional well-being and needs over their own fears and challenges. They mentioned:

‘I felt very responsible for my staff at the time, so I felt like I had to keep them safe.’ (MMP4)

‘I had to focus on my team and how they’re feeling and what they’re dealing with rather than what I am going through and the fear. I didn’t have time to sit and fear for myself, my life and my family. So, for me, I was just focusing on the team. And it helped me deal with what I was going through because I had something else to focus on.’ (MMP5)

The participants in this study described how they prioritised the needs of their staff members above their own, accepting the burdens of the team while quietly managing their own emotions.

### Theme 3: Managerial growth amidst the pandemic

Diagnostic radiography middle managers in this study highlighted that the challenges they faced helped them to build resilience, adaptability, strong leadership and interdisciplinary engagement skills, which contributed to their growth as managers.

#### Category 3.1: Fostering teamwork and collaboration

Diagnostic radiography middle managers shared that staff members worked together more closely and ensured everyone, especially junior staff, followed the protocols. The DRMM team held brainstorming sessions, WhatsApp group chats, and regular online meetings to seek guidance, share vulnerabilities, and learn from each other. Some DRMM responses were:

‘The reality of it is that we got through it because of being able to rely on your own group of your team and also, you know, just your other managers, your other fellow managers that were in the same struggle.’ (MMP3)

‘As a team of middle managers we could brainstorm and we could put ideas to the table and during the Teams meetings and the check-ins that we had it really gave me the platform to assess where I am personally within my department, where do I have any shortcomings and from that platform, so that was a great coping mechanism.’ (MMP9)

The DRMMs in the current study found that communication channels during the pandemic not only promoted information sharing among staff members but also served as a platform for peer support. Diagnostic radiography middle managers also felt that the pandemic fostered great teamwork and cohesion, and mentioned that regular team engagements encouraged staff members to share valuable suggestions.

#### Category 3.2: Interdisciplinary support

The radiology practice where the study was conducted operates across multiple hospital groups; therefore, DRMMs were required to engage regularly with other healthcare professionals and hospital staff. Diagnostic radiography middle managers indicated that they received great support from the staff members at the different hospital groups, with some DRMMs being included in the hospital response teams and disaster management teams. They shared:

‘For the first time, you had weekly meetings with the hospital management about what was going on during the pandemic, what they implemented on their side, and what protocols we implemented. We had a focus group, part of the actual disaster management team, and I was included in that group.’ (MMP8)

‘Also there were regular occupational health and safety meetings which I had to attend. The Covid task team, which I had to attend once a week.’ (MMP11)

The participants in this study reported receiving support from various hospital staff. This included being included in the hospital disaster task force teams, attending regular infection control meetings, and maintaining regular contact with relevant hospital stakeholders.

#### Category 3.3: Personal and professional growth

The DRMMs felt they made notable improvements in their management skills, especially in communication, decision-making, handling pressure, resilience, adaptability and crisis management. They had to be resourceful and learn on their own, which helped them grow personally during that time:

‘We were stretched in that period. It was a lot of unknowns for us; I mean, that was uncharted territory for us as middle managers. Even the way we are now communicating, Zooming and Teams meetings. So that in itself is something that we have learnt. That’s new. A lot of what we’re doing now is on Teams. A lot of our spreadsheets and all sorts of things are on Teams.’ (MMP3)

‘The biggest impact that it had on me was the people aspect. I picked up such amazing skills of listening with understanding and not interrupting people when they talk and just letting them speak.’ (MMP5)

Upon reflecting on their experiences during the COVID-19 crisis, DRMMs shared growth developments, particularly people skills such as learning how to listen to their employees with understanding, communicating constantly and honestly, educating themselves on the unknown topic of COVID-19, as well as skills development to assist in their administrative role as managers.

## Discussion

The key findings of this study were that: (1) DRMMs experienced many challenges during this pandemic crisis period, (2) they were stressed and unsure how to handle the many uncertainties and (3) it was discovered that these opportunities facilitated both professional and personal development. Diagnostic radiography middle managers indicated that the enormity of the tasks at hand was overwhelming at first, and they felt unprepared, disorganised and ill-equipped to manage the challenges. Every scenario they experienced was the first of its kind, and they had to learn in the moment how to handle these situations. Diagnostic radiography middle managers indicated they were expected to implement all the newly communicated guidelines and had nothing at hand to refer to. They had to navigate through the challenges of the pandemic while working to make this happen. By having a crisis management manual available in the radiology department, future DRMMs will have a guide for the important aspects of crisis management and how to implement them. The WHO Health Emergency and Disaster Risk Management Framework report advised that it is important that information collection, analysis and dissemination be harmonised and mechanisms put in place to ensure that ‘the right information gets to the right people at the right time’ across relevant sectors (WHO Health Emergency and Disaster Risk Management Framework report 2019:10). Diagnostic radiography middle managers were thrust into a rapidly changing environment, with policy, staffing, workflow and infection control procedures constantly evolving. They had to continuously adapt, aligning with theme one: Adapting to Change. It is therefore proposed that, as part of the induction process for all employees, the crisis management manual be formally introduced to all radiology staff members. Furthermore, it should be accessible on the radiology practice intranet, where staff members can access it. The availability of this manual will allow all radiology staff members to familiarise themselves with the department’s procedures, policies and protocols, promoting preparedness during a crisis period. Future management can utilise platforms such as the Google Forms application to conduct online knowledge assessments with staff members as part of frequent compliance checks. This can also create a culture of continuous learning and keep employees engaged with practice protocols and guidelines, and prepared for any crisis period. The DRMMs mentioned that they did not know how to handle the stress and anxiety experienced by staff members, especially the alternative financial measures put in place by the radiology practice management, considering the decline in imaging operations, which resulted in cuts in salaries to staff because of low patient numbers, unpaid leave days and no overtime payment. They mentioned feeling mentally fatigued, and some mentioned struggling to handle the stress and found it difficult to express their emotions. They felt they had to stay calm and in control, be supportive of their staff members and thus prioritise the needs of staff members over their own. The financial difficulties they and their staff members experienced because of the cost-cutting measures weighed heavily on them, as they knew staff depended on them for support and guidance. They realised that their reactions to situations affected how staff members adapted to the challenging environment, so they felt the need to hide their emotions. As advised by the WHO Health Emergency and Disaster Risk Management Framework report, key steps in developing strategies for implementing priority actions should include components for health security, mental health and disease surveillance to address prevention, emergency preparedness, response and recovery (The WHO Health Emergency and Disaster Risk Management Framework report 2019:12). The researcher believes that establishing a good departmental employee well-being system in any organisation can be beneficial to all employees. Financial tutorials on unpredictable situations can also benefit staff, many of whom may be unprepared for their financial implications. Smith and Friedman (2022:10) assert that encouraging employees to establish an emergency financial fund is crucial for preparing for unprecedented times. These findings align with theme two: Challenging Environment, which demonstrates the emotional strain, financial instability, fear and moral obligation managers felt to care for their staff during crisis conditions. The employee well-being system should be widely communicated and accessible to all employees, with the information of the wellness councillor visible in the department break rooms and practice intranet for easy access to employees and to eliminate the societal stigma of utilising these services. Employees should be allowed to utilise this service when needed and without incurring any expense. Managers should also pre-emptively prepare staff members for financially difficult times by providing them with financial information sessions from financial advisors, either in group sessions or individual counselling. This can be conducted in-person or virtually. Managers can cultivate a cohesive team environment where everyone feels supported and safe by structured peer-to-peer discussions where staff members can be randomly paired with a colleague and where they must provide supportive, positive feedback and observations about each other. The present study considers that such an initiative will cultivate a culture of trust, foster teamwork and promote positive interpersonal relationships among colleagues. Diagnostic radiography middle managers were enriched by a network of support structures, which depended on building professional relationships by seeking guidance and knowledge sharing, as well as teamwork. Participants shared that they had to educate themselves, including how to swiftly use technological applications. The DRMMs reported that their experiences as managers during the pandemic contributed significantly to improvements in their managerial skills. They reported that their interpersonal relationships with their teams, as well as hospital colleagues, were improved. The measures they took to ensure the management controls remained effective and functioning optimally contributed to the successful navigation of their teams through this pandemic. The healthcare managers in a study by Uvhagen et al. (2024:363) stated that they recognised more than before that to give adequate care for all patients, the whole healthcare system needed to work ‘as one’, instead of in ‘silos’. As described by Nembhard, Burns and Shortell (2020:4), when facing novel circumstances, organisations thrive by leveraging both internal and external relationships. The DRMMs in this study received support from hospital staff through involvement in disaster task force teams, regular infection control meetings and contact with relevant stakeholders. Yasar et al. (2017:8) acknowledged that the dimensions of management skill and self-management have positive and significant relationships with organisational performance. Organisations must furnish leaders such as DRMMs with all the requisite tools and support to deliver essential services for uninterrupted operations, particularly during periods of overwhelming and challenging circumstances. As described by Vu (2020:20), empowerment fosters employee creativity, quality of work-life, spirit of teamwork and organisational effectiveness. Engaging in employee empowerment strategies and techniques provides a feeling of support, an increase in confidence and employees will demonstrate a high level of commitment if they recognise that top management displays commitment to provide adequate resources (Vu 2020:22). The challenges experienced by DRMMs required them to develop new strategies for communication, implement operational changes, and educate their teams effectively. Diagnostic radiography middle managers also mentioned how they had to learn how to conduct virtual online meetings and use an Excel spreadsheet as they had to document data. These practices can be taught to DRMMs in training sessions. They shared that conversations and meetings with peers helped them cope and apply workable methods in their own teams, and they could ask for guidance from their fellow DRMMs if they struggled with complicated situations. The regular interactions between DRMMs and hospital departments led to greater tolerance of one another in handling difficult situations. It fostered meaningful working relationships and created a sense of harmony that could potentially filter down to the different teams. The findings demonstrate how the pandemic acted as a catalyst for growth, strengthening teamwork, leadership competencies and professional relationships, as indicated in theme three: Managerial Growth Amidst the Pandemic. In times of crisis, as described by Everly et al. (2020:768), leaders can establish routines by scheduling daily briefings to share new information electronically. Managers and leaders should get training in the basic principles of ‘psychological first aid’ (Everly et al. 2020:768). Kolbe and Broos (2019:4) found that teams learn best by reflecting on their work, sharing expertise, problem-solving and decision-making procedures that suit the task. O’Doherty et al. (2020:6) suggested that institutions should share leadership techniques to help prepare for future pandemics, such as clear and concise communication of necessary information, gratitude for frontline staff and compassion for personnel during this stressful time. Paulsen (2020:872) explained that when people are reflective, they can enrich or limit their experience. Diagnostic radiography middle managers reported that the pandemic offered valuable learning opportunities. Through reflection, DRMMs acknowledged the necessity of staying informed about evolving guidelines and best practices, through collaborations and group reflections, to support their staff during periods of uncertainty. Therefore, this study suggests that structured best practice meetings, workshops and reflection sessions can be established to provide DRMMs with dedicated sessions to share experiences, seek guidance on challenging situations, and learn from one another. To assist DRMMs with leadership strategies during a pandemic, they could be provided with leadership and crisis management training.

### Strengths, limitations and recommendations

The study highlighted the experiences of DRMMs during the COVID-19 pandemic, which encompassed a confluence of significant challenges and uncertainties, concurrently fostering substantial personal and professional development. Their experiences accentuated their resilience, strength and enhanced confidence they developed in their managerial skills. The study was conducted at one private radiology practice, which excluded all the other private practices in the study region. As a result, the findings may not reflect the broader experiences of DRMMs across the private and government sectors. The findings of the study may not be fully generalisable to all DRMM experiences, as it assumes a shared set of experiences and challenges and does not account for individual experiences.

A few recommendations developed from the unique circumstances DRMMs faced during the pandemic that could enhance management practices to benefit radiology practices in following best practices in a crisis would be:

- A crisis management document should be introduced in radiology departments to facilitate centralised communication channels, standard operating protocols and support DRMMs in adopting new working methods.
  ■ *Channels to communicate policies and/or operations-related matters to teams should be established*.
  ■ *Ongoing managerial education and staff compliance checks should be implemented*.
- Radiology departments should have an employee health and wellness programme in place to assist employees with emotional and psychological support.
  ■ *Provide access to employee mental health support programmes*.
  ■ *Provide guided financial wellness planning sessions*.
  ■ *Cultivate a cohesive team environment*.
- Diagnostic radiography middle managers should participate in interdisciplinary team meetings and collaborations to form meaningful relationships with other teams. Diagnostic radiography middle managers can also provide interdisciplinary teams with presentations about radiography to provide insights into radiography operations and important radiography protocols.
  ■ Establishment of regular interdisciplinary team meetings.
- It is also recommended that regular, structured reflection and debriefing sessions be facilitated for DRMMs during and after major crises to facilitate shared learning, emotional processing and professional growth in a calm and supportive environment.
  ■ *Sharing of best practices for handling pandemic situations*.
  ■ *Encouragement of reflective and debriefing sessions*.

## Conclusion

During this study, DRMMs faced many operational management factors, which were supported by verbatim quotations during the semi-structured interviews, resulting in an understanding of the experiences of these DRMMs. The DRMMs shared that their experiences during the coronavirus pandemic brought about meaningful changes in how they viewed themselves and their roles within the workplace. They felt they made significant improvements in their management capabilities, particularly in areas such as communication skills, decision-making, working under pressure, resilience, adaptability and an understanding of crisis management. They indicated that they had to be resourceful and find out information on their own, contributing to their overall personal development during a crisis period. They shared that as the pandemic progressed and they found their way around handling challenging situations, they found joy in the personal and professional growth they experienced. Ultimately, their experiences suggest that the skills and resilience developed during the pandemic will enable them to manage future health crises more effectively and with greater confidence.
